# Comparative assessment of the quality of age-at-event reporting in three HIV cohort studies in sub-Saharan Africa

**DOI:** 10.1136/sti.2008.033423

**Published:** 2009-03-13

**Authors:** A Wringe, I Cremin, J Todd, N McGrath, I Kasamba, K Herbst, P Mushore, B Żaba, E Slaymaker

**Affiliations:** 1 London School of Hygiene and Tropical Medicine, London, UK; 2 Imperial College, London, UK; 3 Medical Research Council/Uganda Virus Research Institute, Uganda Research Unit on AIDS, Entebbe, Uganda; 4 Africa Centre for Health and Population Studies, University of KwaZulu-Natal, South Africa; 5 Biomedical Research and Training Institute, Harare, Zimbabwe

## Abstract

**Objectives:**

To assess inconsistencies in reported age at first sex (AFS) and age at first marriage (AFM) in three African cohorts, and consider their implications for interpreting trends in sexual and marital debut.

**Methods:**

Data were analysed from population-based cohort studies in Zimbabwe, Uganda and South Africa with 3, 10 and 4 behavioural survey rounds, respectively. Three rounds over a similar time frame were selected from each site for comparative purposes. The consistency of AFS and AFM reports was assessed for each site by comparing responses made by participants in multiple surveys. Respondents were defined as unreliable if less than half of all their age-at-event reports were the same. Kaplan-Meier functions were used to describe the cumulative proportion (1) having had sex and (2) married by age, stratified by sex, birth cohort and site, to compare the influence of reporting inconsistencies on these estimates.

**Results:**

Among participants attending all three comparable rounds, the percentage with unreliable AFS reports ranged from 30% among South African women to 56% among Zimbabwean men, with similar patterns observed for AFM. Inclusion of unreliable reports had little effect on estimates of median age-at-event in all sites. There was some evidence from the 1960–9 birth cohort that women in Uganda and both sexes in South Africa reported later AFS as they aged.

**Conclusion:**

Although reporting quality is unlikely to affect comparisons of AFS and AFM between settings, care should be taken not to overinterpret small changes in reported age-at-event over time within each site.

Data on trends in age at first sex (AFS) and age at first marriage (AFM) in African settings are important for understanding the epidemiology of HIV and for informing and evaluating HIV prevention strategies, as well as for understanding trends in fertility and teenage pregnancy. Several recent studies have documented trends in AFS and AFM in African settings,^1^^–^5 often analysing data from multiple rounds of Demographic and Health Surveys (DHS) which are undertaken every few years in many developing countries. However, as noted by Żaba *et al*, these studies often exhibit methodological problems which may introduce bias.^6^ These include a failure to allow for age censoring, or to consider differences in age structure when comparing crude proportions, or to include both current status data and recall data. Furthermore, changes in the population being surveyed due, for example, to migration or mortality may make it difficult to distinguish between real changes in the age-at-event being monitored, the effects of biased reporting and reporting errors and the role of confounding variables when interpreting trends over time.^7^

Previous studies investigating the quality of self-reported age-at-event reports have used various methods including assessing the consistency of AFS against other indicators such as age at first birth, or by comparing behavioural data with biological outcomes such as sexually transmitted infections and pregnancy.^4^ ^8^^–^10 Other approaches have included conducting internal survey validity checks by comparing age-at-event reports among a subsample of re-interviewed survey participants,^11^ and comparing AFS reports from different surveys among similar populations.^7^ Finally, data quality in DHS surveys has been assessed by comparing median AFS across multiple cross-sectional surveys for the same birth cohort.^6^

These assessments have identified a range of issues relating to reporting quality, including inconsistent reporting of the same event among re-interviewed participants, under-reporting of ever having had sex among young unmarried respondents, inconsistencies between AFS and age at first birth, and evidence of changes over time in reported age of sexual debut by the same birth cohort.^6^ ^9^^–^11

There are several reasons why inaccurate reporting of AFS and AFM may occur in different settings. First, questions about sexual debut are sensitive in nature, which can result in social desirability bias.^8^ Some studies have observed that women tend to under-report their sexual activity, with the reverse being true for men, possibly reflecting gender-specific social norms and expectations about sexual debut.^1^ ^6^ ^12^ Social desirability bias is also likely to be a function of exposure to HIV prevention messages, which have encouraged delayed onset of sexual initiation and fewer sexual partners following sexual debut.^13^ Reporting quality of sexual and marital debut will also depend on how accurately people recall the date of these events. Recall bias resulting in inaccurate reporting of AFS and AFM may be most common among older people due to the longer average time since the event occurred. Furthermore, the extent of recall bias may differ for reported AFS and reported AFM among older people, depending on whether sexual debut or marriage is a more memorable event.

Longitudinal studies that repeatedly ask the same questions to a study population over sequential survey rounds offer an opportunity to assess reporting inconsistencies in age-at-event reports. By comparing the extent and direction of inconsistencies in reported ages of sexual and marital debut within and between sites, it is possible to assess the effect of bias on documented trends in age at these events and to determine whether these biases are of a similar magnitude across different settings. This paper describes reporting quality and assesses the extent and influence of reporting bias in AFS and AFM in cohort studies in Zimbabwe, Uganda and South Africa in order to assist interpretation of trends in marital and sexual debut in these populations.

## METHODS

### Data sources

Three HIV cohort studies participating in the ALPHA network^14^ were selected for this analysis because they had completed at least three survey rounds which included questions on AFS and AFM. In the Manicaland cohort in eastern Zimbabwe, three survey rounds have been undertaken between 1998 and 2005. In the Masaka cohort in south-western Uganda, behavioural questions have been included in 10 annual survey rounds since 1996 and, in the Africa Centre Demographic Information System (ACDIS) in Umkhanyakude district in KwaZulu Natal, South Africa, four behavioural survey rounds have been completed between 2003 and 2007. Detailed descriptions of the setting and of the study methods used at each of these sites is provided in previous publications^15^^–^17 and in the site-specific analyses of marriage and sexual debut that are presented elsewhere in this supplement.^18^^–^20 For this analysis, each site provided data on participant identifiers and variables describing the dates of rounds attended, date of birth, sex, whether the participant reported having ever had sex and having ever married and, if applicable, their recalled age at first sex and recalled age at first marriage at each round attended.

Table 1 summarises the dates and key characteristics of the survey rounds and participants at each site. The proportion of respondents with consistent age-at-event reports in each site will be influenced by the number of rounds in which they have participated, with a higher number of rounds resulting in a lower proportion of consistent responses. Therefore, in order to compare reliability of reported AFS and AFM across sites, three rounds were selected from each study, with similar spacing between rounds and covering approximately the same time frame (table 1).

**Table 1:** Characteristics of survey rounds and participants at each study site

	Site		
	Manicaland	Masaka	Umkhanyakude
Number of rounds	3	10	4
Frequency of rounds	2–3 years	Annual	Annual
Age range of participants (years)	M:17–54, F:15–44*	M:13+, F:13+	M:15–54, F:15–49
Total number of male respondents	10096	7316	13315
Total number of female respondents	13421	8484	20231
Year of first round (round number)	1998–2000 (1)	1996–1997 (1)	2003–2004 (1)
Year of last round (round number)	2003–2005 (3)	2005–2006 (10)	2007 (4)
Rounds used for comparative analysis§	1, 2, 3	4, 7, 10	1, 2, 4
Participation in comparative rounds†	79%, 79%, 83%	65%, 64%, 61%	58%, 66%, 48%
% attending 1 of 3 comparative rounds‡	M:73%, F:70%	M:57%, F:55%	M:69%, F:60%
% attending 3 of 3 comparative rounds‡	M:13%, F:18%	M:16%, F:20%	M:5%, F:11%

*Male and Female: 15–54 in round 3.

†Denominator defined as eligible for each round.

‡Denominator defined as participated in 1+ comparative round.

§For analyses requiring similar time frame and round spacing.

### Definitions

#### Reporting consistency

Using age-at-event reports from each survey round and the definitions shown in table 2, participants were categorised as either (1) consistent reporters, (2) inconsistent reporters for whom age-at-event can be corrected, (3) inconsistent reporters for whom age-at-event can be estimated or (4) unreliable reporters.

**Table 2:** Definitions of reliable and unreliable reporting

Category	Number of age-at-event reports			Imputed response	
	1	2	3+		
Consistent	Report ⩽ age at survey	Both reports same AND both reports ⩽ age at survey	All reports same AND all reports ⩽ age at survey	Reported age	Reliable
Inconsistent: can be corrected	N/A	Reports differ by 1 year AND both reports ⩽ age at survey	2 ages reported which differ by 1 year AND both reports ⩽ age at survey	Older of the 2 ages	Reliable
Inconsistent: can be estimated	N/A	1 report ⩽ age at survey	⩾50% of reports same AND these reports ⩽ age at survey	Most frequent report that is ⩽ age at survey	Reliable
Unreliable: cannot be estimated or corrected	Report >age at survey	Reports differ by >1 year AND both reports ⩽ age at survey OR both reports > age at survey	<50% of reports same OR ⩾50% reports same AND reports > age at survey	Mean of all reported ages	Unreliable

In brief, consistent reporters were those who only reported once or for whom all age-at-event reports were the same, if these ages were younger than or the same as age at interview. For respondents with inconsistent reports, the age-at-event was corrected if two different ages were reported which were 1 year apart and both ages were younger than or the same as, age at interview. In this case, the report was corrected to the older of the two reported ages. Individuals were defined as inconsistent reporters for whom an age-at-event could be estimated if at least half of all reported ages were the same and were younger than or the same as age at interview. Respondents were defined as unreliable if less than half of all age-at-event reports were the same or reports differed by more than 1 year, or if at least half of all age-at-event reports were the same but were older than ages at interview. Consistent reporters and inconsistent reporters for whom age-at-event could either be estimated or corrected were defined as reliable. For survival analyses that included unreliable reporters, age at sexual or marital debut was defined as the mean of all reported event ages.

#### Marriage

Definitions of marriage varied between the three sites and are described in detail elsewhere.^21^ The definition of marriage used in Manicaland included cohabitation or a relationship lasting longer than 6 months (round 1) or 12 months (rounds 2 and 3) as well as civil and traditional marriages, in contrast to Masaka and Umkhanyakude where cohabitation and long-term partnerships were not included.

### Statistical analysis

All statistical analyses were carried out using Stata version 10 (Stata Corp, College Station, Texas, USA). All analyses were stratified by sex, since patterns of sexual and marital debut as well as behavioural reporting biases differ between the sexes.^6^ ^12^

The consistency of AFS and AFM reporting within each site was assessed by comparing responses made by non-virgin or ever married participants using data from all available rounds. Differences in reporting quality between sites were assessed by considering participants who had attended all three rounds selected for the comparative analysis and comparing the distribution of participants across the four consistency categories. Tabulations and χ^2^ tests were used to compare the distribution of AFS and AFM reports and consistency of AFS and AFM reports in each site.

Survival analysis was used to estimate the distribution of AFS and AFM from censored observations^6^ among participants attending any of the three comparable rounds. The input data were age of the respondent, whether or not they had ever experienced the event being assessed (ie, either sexual debut or first marriage) and, if applicable, recalled AFS or AFM.

Survival functions were calculated to describe the probability of remaining a virgin or of remaining unmarried by age, using reported AFS or AFM as the failure event and censoring those who never had intercourse or who had never married at their age at the time of the survey. In order to smooth the survival functions we added a randomly selected fraction between 0 and 1 to each report of AFS and AFM. The median AFS and AFM and their corresponding interquartile ranges were then reported from these survival functions to summarise the distribution of age at each event. In order to investigate whether reporting among the same individuals changed as time from the event increased, survival analyses were stratified by 10-year birth cohorts, with the earliest common birth cohort defined as participants born between 1950 and 1959 and the latest birth cohort consisting of those born between 1980 and 1989.

Trends over time in AFS and AFM reporting were examined by considering the median age for each event for the 1960–9 birth cohort in each site among those who attended any of the three rounds selected for the comparative analysis. This mid-range birth cohort was selected so the event being reported was neither too recent (in which case changes in reporting may not yet have reached their full extent) nor too distant in time (in which case it may be too late to detect any reporting changes). As well as the comparative analyses using the three selected rounds, additional analyses were conducted, where appropriate, to further explore the extent of reporting changes in each site using the full range of rounds.

## RESULTS

### Response rates to AFM and AFS questions, by site and sex

The total number of male and female respondents who participated in one or more of all survey rounds in each site is shown in table 1. In Manicaland, 75% of men and 77% of women reported ever having sex, while the corresponding percentages were 62% and 72% in Masaka and 59% and 74% in Umkhanyakude. A lower percentage of men than women reported having ever married in all sites: 47% vs 72% in Manicaland, 42% vs 58% in Masaka and 8% vs 13% in Umkhanyakude (reflecting the particularly low marriage rates in South Africa).^21^ ^22^

### Consistency of AFS and AFM reporting

#### Within-site comparisons

The consistency of AFS and AFM reporting was compared within each site using data from all surveys for individuals who had reported either or both events. In Manicaland the proportion with unreliable AFS reports was similar to the proportion with unreliable AFM reports for men (∼50%) as well as for women (∼30%). In Masaka the proportion with unreliable AFS reports was higher than the proportion with unreliable AFM reports (50% vs 41% for men and 34% vs 28% for women) while, in Umkhanyakude, the proportion with unreliable AFS reports was lower than the proportion with unreliable AFM reports (43% vs 52% for men and 29% vs 35% for women).

#### Between-site comparisons

**Figure 1:**
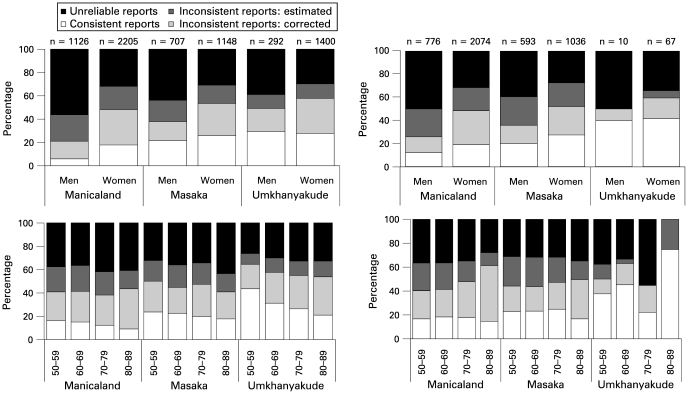
Quality of age at first sex (AFS) reports (left panels) and (B) age at first marriage (AFM) reports (right panels) across sites among non-virgin and ever married subjects attending all three comparable survey rounds, by sex in row 1 and by birth cohort in row 2.

Table 1 shows the participation rates among the eligible population in the three survey rounds selected for comparison between sites and the percentage of participants attending all three of the rounds selected for the comparison. Among those attending all three of the comparative rounds, the patterns in reporting quality between the sexes were similar for AFS and AFM reporting, with a higher proportion of inconsistent reporters among men than among women in all three sites for both events (fig 1). In relation to AFS reporting among men, the proportion with unreliable reports was lowest in Umkhanyakude (39%) and highest in Manicaland (56%), while among women the proportion of unreliable responses was similar between sites at almost one-third. In comparison, for AFM the proportion of unreliable reporters among men ranged from 39% in Masaka to 50% in Manicaland and Umkhanyakude, and the proportion of unreliable women ranged from 27% in Masaka to 34% in Umkhanyakude.

In relation to reporting consistency of AFS by birth cohort, the proportion of unreliable respondents increased slightly among later birth cohorts in all sites, with this being most apparent in Masaka and Umkhanyakude (fig 1). A similar result was seen for AFM reporting in Masaka and to a lesser extent in Umkhanyakude, where the number of ever married persons in the later birth cohorts was small. In Manicaland the proportion of participants with unreliable AFM responses was lowest in the latest birth cohort (fig 1). In all sites, <2% of unreliable respondents were classified as unreliable due to reporting ages at event which were older than their age at survey.

### Influence of unreliable reporting on estimates of AFM and AFS

Median AFS and AFM for reliable and unreliable reporters by birth cohort, site and sex are shown in table 3. Overall, inclusion of unreliable reporters had little effect on estimates of median AFS and AFM. However, among the earliest birth cohort, the median AFS was slightly older among unreliable reporters than for those who were reliable, with the exception of women in Umkhanyakude and men in Masaka. For the latest birth cohort, the inclusion of unreliable reporters resulted in a younger median AFS among men in Manicaland and Umkhanyakude but did not affect the estimate of median AFS among women in any of the sites.

**Table 3:** Median age at first sex (AFS) and age at first marriage (AFM) in each site by reliability of data among those attending the three comparative rounds

Site	Cohort	Indicator	All data	Reliable data	Unreliable data
			Median (IQR)	Median (IQR)	Median (IQR)
*Men*					
Manicaland	1950–9	AFS	20.1 (18.3–22.5)	19.9 (18.1–22.2)	20.8 (19.2–23.0)
		AFM	24.7 (22.2–27.2)	24.9 (22.2–27.4)	24.2 (22.5–26.9)
	1960–9	AFS	19.6 (17.9–21.7)	19.4 (17.7–21.8)	19.9 (18.3–21.4)
		AFM	25.0 (22.3–27.9)	25.3 (22.3–28.2)	24.0 (22.0–26.5)
	1970–9	AFS	19.1 (17.4–21.2)	19.1 (17.3–21.3)	19.0 (17.6–20.8)
		AFM	24.8 (22.3–27.5)	25.1 (22.6–27.9)	22.3 (20.5–23.8)
	1980–9	AFS	19.2 (17.4–21.4)	19.4 (17.5–21.6)	17.5 (16.6–18.9)
		AFM	24.3 (22.3–25.7)	24.3 (22.5–25.2)	20.2 (18.7–22.1)
Masaka	1950–9	AFS	18.3 (17.1–20.0)	18.4 (17.3–20.0)	17.8 (16.8–19.9)
		AFM	23.3 (20.8–26.7)	23.0 (20.6–26.0)	24.9 (22.7–27.9)
	1960–9	AFS	18.1 (17.0–19.5)	18.0 (17.0–19.0)	18.6 (17.3–20.4)
		AFM	23.0 (20.6–26.3)	23.0 (20.3–26.0)	23.7 (21.7–26.6)
	1970–9	AFS	18.0 (16.6–19.0)	18.0 (16.5–19.0)	17.6 (16.7–19.5)
		AFM	23.9 (21.0–27.3)	24.4 (20.9–28.1)	22.4 (21.3–24.2)
	1980–9	AFS	16.7 (15.3–18.0)	16.7 (15.3–18.0)	16.9 (15.5–17.7)
		AFM	24.6 (21.8–27.6)	25.2 (21.8–)	21.3 (19.5–23.2)
Umkhanyakude	1950–9	AFS	20.2 (18.0–22.3)	20.2 (17.9–22.1)	20.5 (18.8–23.3)
		AFM	– (38.6–)	– (40.4–)	32.5 (27.1–41.7)
	1960–9	AFS	19.3 (17.6–21.5)	19.2 (17.4–21.2)	20.0 (18.4–22.6)
		AFM	– (41.8–)	– (42.6–)	31.1 (27.6–34.9)
	1970–9	AFS	18.3 (16.5–20.3)	18.3 (16.4–20.2)	18.8 (17.2–20.5)
		AFM	– (–)	– (–)	32.1 (28.6–32.5)
	1980–9	AFS	17.1 (15.9–18.6)	17.2 (15.9–18.7)	17.0 (15.7–18.1)
		AFM	– (–)	– (–)	– (–)
					
*Women*					
Manicaland	1950–9	AFS	19.0 (17.2–20.7)	19.0 (17.1–20.6)	19.6 (18.0–21.3)
		AFM	19.3 (17.6–21.0)	19.1 (17.5–20.8)	20.2 (18.4–21.8)
	1960–9	AFS	18.7 (17.0–20.6)	18.7 (17.0–20.6)	18.7 (17.4–20.7)
		AFM	19.0 (17.4–21.1)	19.0 (17.2–21.0)	19.4 (17.9–21.5)
	1970–9	AFS	19.0 (17.5–21.0)	19.0 (17.5–21.0)	18.5 (17.2–20.4)
		AFM	19.7 (17.9–21.9)	19.8 (18.0–22.0)	19.1 (17.5–20.7)
	1980–9	AFS	19.0 (17.6–20.6)	19.0 (17.6–20.7)	17.8 (16.9–18.8)
		AFM	19.6 (18.0–21.5)	19.7 (18.1–21.6)	18.1 (17.1–19.1)
Masaka	1950–9	AFS	16.7 (15.5–18.0)	16.5 (15.4–18.0)	17.2 (16.0–18.6)
		AFM	18.0 (16.1–19.9)	17.9 (16.0–19.3)	18.5 (17.4–20.8)
	1960–9	AFS	16.9 (15.7–18.3)	16.9 (15.6–18.3)	17.1 (16.2–18.1)
		AFM	18.2 (16.8–20.1)	18.1 (16.4–19.9)	19.1 (17.7–20.8)
	1970–9	AFS	17.0 (15.9–18.4)	17.0 (15.8–18.4)	17.2 (16.3–18.6)
		AFM	18.6 (17.0–20.8)	18.5 (17.0–20.7)	18.8 (17.2–21.5)
	1980–9	AFS	16.8 (15.8–18.0)	16.8 (15.7–18.0)	16.8 (16.0–17.9)
		AFM	19.2 (18.0–21.4)	19.4 (18.1–21.6)	18.1 (17.3–19.9)
Umkhanyakude	1950–9	AFS	18.6 (17.0–20.3)	18.6 (17.0–20.3)	18.5 (17.3–19.9)
		AFM	– (41.9–)	– (48.4–)	25.9 (24.4–29.3)
	1960–9	AFS	18.6 (17.0–20.3)	18.5 (16.9–20.4)	19.0 (17.4–20.6)
		AFM	– (31.4–)	– (34.0–)	28.9 (24.5–32.1)
	1970–9	AFS	18.3 (17.3–20.6)	18.8 (17.3–20.6)	19.1 (17.5–20.5)
		AFM	– (38.5–)	– (–)	25.3 (23.0–29.0)
	1980–9	AFS	18.0 (17.0–19.2)	18.0 (16.9–19.2)	17.9 (17.1–19.2)
		AFM	– (–)	– (–)	24.7 (20.4–26.8)

A similar pattern was observed for AFM where, for the earliest birth cohorts, the median AFM was slightly older among unreliable reporters than among those who were reliable for women in Manicaland and both sexes in Masaka. In the latest birth cohort the median AFM was younger among unreliable reporters than among those who were reliable for all sites where it was possible to calculate a median AFM.

### Trends in AFS and AFM reporting over time

Table 4 shows the median AFS and AFM for individuals born in 1960–9 among those who attended any of the rounds used in the comparative analysis. There was a tendency among men and women in Masaka and Umkhanyakude to report older AFS over the three rounds, but little change in AFS reporting among men and women in Manicaland. There was some evidence that men were reporting younger AFM over time in Manicaland and Masaka, with little change observed among women in these sites.

**Table 4:** Trends in median age at first sex (AFS) and age at first marriage (AFM) over three selected rounds with a comparable time frame among those born 1960–9

Indicator	Site	Sex	First round	Middle round	Last round
AFS	Manicaland	Men	19.4	19.8	19.4
		Women	18.6	18.6	18.7
	Masaka	Men	18.0	18.2	18.2
		Women	16.3	16.8	16.8
	Umkhanyakude	Men	18.8	20.0	20.0
		Women	18.5	18.5	18.8
AFM	Manicaland	Men	25.9	23.4	25.0
		Women	19.2	18.9	19.1
	Masaka	Men	23.8	22.9	22.8
		Women	18.2	18.5	18.3
	Umkhanyakude*	Men	–	–	–
		Women	–	–	–

*Insufficient marriage reports in Umkhanyakude to calculate median AFM.

For Masaka, where annual surveys have been conducted over a 10-year period, trends in AFS and AFM were also calculated using the first, middle and last of the 10 completed rounds. Over this longer time frame the median reported AFS among women in the 1960–9 birth cohort increased from 15.8 years in 1996–7 to 16.5 years in 2005–6 while, among men, it remained consistent at 18.0 years over the same period.

**Figure 2:**
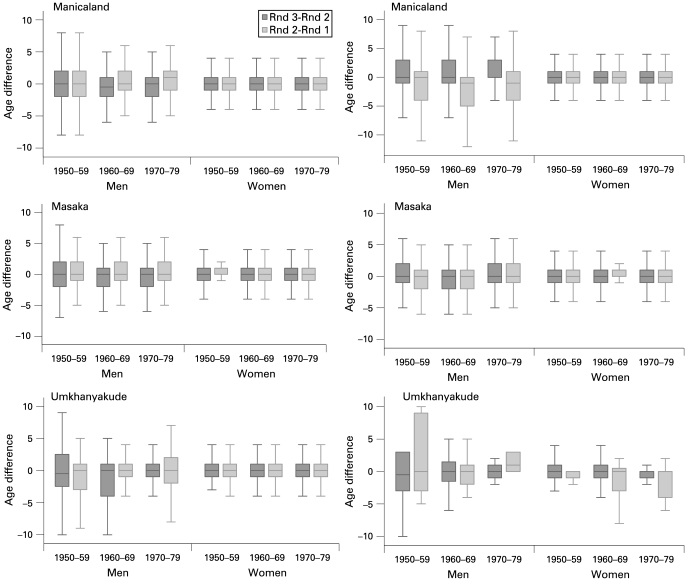
Differences in reported age at first sex (AFS) (left panels) and reported age at first marriage (AFM) (right panels) between consecutive comparison rounds by birth cohort, sex and site among those who reported in all three of the comparative rounds. The box plots show the interquartile range of values for this difference with the horizontal line within each box representing the median difference and the external vertical lines signifying the maximum and minimum reported age difference within each category.

Figure 2 shows the differences in reported age-at-event between each of the rounds used for the comparative analysis by sex, birth cohort and site among those who had reported in all three of the comparative rounds. For both AFS and AFM, these figures show that, despite considerable differences in reported age-at-event between the three comparative rounds (particularly among men), the median difference in reported ages was consistently close to zero for both sexes.

## DISCUSSION

Overall, this study shows that inconsistent reporting of age at sexual and marital debut is a common phenomenon in these sites, with over one-third of individuals who participated in three comparative survey rounds defined as unreliable in relation to reporting of AFS and AFM. Nevertheless, despite the high levels of inconsistent reporting observed and the range of the differences in reported AFS and AFM in sequential comparison rounds, the median difference between reported ages at event was consistently close to zero. Indeed, the inclusion of inconsistent reporters using the mean of all reported ages tended to make little difference to estimates of median AFS or median AFM, particularly among women.

The findings from these analyses also suggested that, although the proportion of unreliable reporters was slightly higher for the latest birth cohorts (particularly for AFS), a high degree of inconsistent reporting occurred among all birth cohorts. Recall bias, which is expected to be increasingly common among the earlier birth cohorts, may work in either direction, possibly explaining why inclusion of unreliable reporters did not result in substantial changes in estimates of median age-at-event compared with their exclusion. Social desirability bias may be a more likely explanation for the discrepancies in age-at-event between reliable and unreliable reporters among those in the later birth cohorts. Although social desirability bias is more likely to work in a single direction, the direction may change over time in the context of ongoing HIV awareness programmes.

**Take-home messages**

- The accuracy of reporting age at first sex (AFS) and age at first marriage (AFM) can be assessed in longitudinal studies with repeat sexual behaviour surveys.
- In studies from South Africa, Zimbabwe and Uganda, inconsistent reporting was common for both AFS and AFM, with men reporting less reliably than women.
- Despite high levels of inconsistent AFS and AFM reporting, inclusion of unreliable reports had little effect on estimated median ages at event.

In Manicaland and Masaka, where marriage rates are high, the proportion of persons with inconsistent reports was higher in relation to AFS than AFM, whereas the reverse was true in Umkhanyakude where a far lower proportion of the population have ever married. The consistency of marriage reporting across the three sites is likely to reflect the way in which marriage questions are asked in each site’s survey rounds, as well as differences in local definitions of marriage. In particular, the higher proportion of unreliable AFM reports in Manicaland compared with Masaka for both men and women may be partially explained by a minor modification to the definition of marriage in the second survey round in Manicaland, as well as the fact that cohabitation is included within the definition of marriage in the Manicaland survey questionnaire but not in the Masaka questionnaire. Not only may relatively short periods of cohabitation be less memorable and therefore less well recalled than ceremonial marriages, but individuals may no longer wish to refer to a period of cohabitation as a marriage if that union is subsequently dissolved, as has been reported in other settings.^12^

Previous studies that have assessed reporting biases in AFS trends in other African studies, as well as in those assessing reports of number of sexual partnerships, have noted that men report earlier ages and women report later ages of sexual initiation than they have experienced.^6^ ^12^ This analysis also identified greater reporting inconsistencies among men than among women in all sites in relation to both marital and sexual debut. Women in the latest birth cohorts with unreliable responses reported similar or older ages of sexual debut than those who were identified as reliable reporters, with the exception of Manicaland. Men born between 1980 and 1989 with unreliable responses tended to report younger ages of sexual initiation than reliable reporters in Manicaland and Umkhanyakude, with the reverse being true in Masaka.

This analysis also suggests that, among the 1960–9 birth cohort, men and women in Umkhanyakude and Masaka were reporting later ages of sexual debut over time, highlighting the need to consider biases when interpreting trends over time in AFS. These findings are consistent with those from other studies which have suggested that apparent changes in reported behaviour may be influenced by changes in social desirability bias.^7^

There are various limitations to this analysis that need to be taken into consideration when interpreting these findings. First, a relatively small proportion of all non-virgin or ever married participants in each of the sites had participated in all three rounds selected for the comparative analysis, particularly among the ever married population in Umkhanyakude where rates of marriage are low. Nevertheless, restricting the analysis to individuals who had participated in all three of these rounds ensured that estimates of consistency across the sites would not be biased by differences in the distribution of the number of rounds attended. Second, trends in reported AFS and AFM may be inaccurate if sexually active or married persons consistently misreport their age at sexual or marital debut. In this analysis we were only able to identify persons who changed their reporting of AFS or AFM in different survey rounds and were unable to detect those who consistently misreport. However, in sites where spouses can be identified within the cohort, future analyses could compare reports of AFM among married couples.

This paper is the first to provide a comparative analysis of both AFS and AFM reports within and between different sites using longitudinal data. While it may be assumed that AFS reporting would be more prone to bias due to its sensitive nature, this analysis suggests that both AFS and AFM indicators are prone to high but similar levels of inconsistent reporting.

In conclusion, these findings show that, despite a high percentage of respondents who report AFS and AFM inconsistently in each of the three sites, unreliable age-at-event reporting only had a moderate effect on estimates of median age-at-event. While reporting quality is unlikely to affect comparisons of age-at-event reports between sites, care should be taken not to overinterpret small changes in reported AFS and AFM over time within each site.
